# Clinical prediction score for superficial surgical site infection after appendectomy in adults with complicated appendicitis

**DOI:** 10.1186/s13017-018-0186-1

**Published:** 2018-06-18

**Authors:** Pinit Noorit, Boonying Siribumrungwong, Ammarin Thakkinstian

**Affiliations:** 1 Department of Surgery, Chonburi hospital, Chonburi, Thailand; 2 Department of Surgery, Faculty of Medicine, Thammasat University Hospital, Pathum thani, Thailand; 3 Section for Clinical Epidemiology and Biostatistics, Faculty of Medicine, Ramathibodi Hospital, Mahidol University, Rama VI road, Rachatevi, Bangkok, 10400 Thailand

**Keywords:** Appendicitis, Surgical site infection, Wound infection, Risk factors, Prediction scores

## Abstract

**Background:**

Superficial surgical site infection (SSI) is common after appendectomy. This study aims to determine a clinical prediction score for SSI after appendectomy in complicated appendicitis.

**Methods:**

Data from randomized controlled trial of delayed versus primary wound closures in complicated appendicitis was used. Nineteen patient- and operative-related predictors were selected in the logit model. Clinical prediction score was then constructed using coefficients of significant predictors. Risk stratification was done by receiver operating characteristic (ROC) curve analysis. Bootstrap technique was used to internal validate the score.

**Results:**

Among 607 patients, the SSI incidence was 8.7% (95% CI 6.4, 11.2). Four predictors were significantly associated with SSI, i.e., presence of diabetes, incisional length > 7 cm, fecal contamination, and operative time > 75 min with the odds ratio of 2.6 (95% CI 1.2, 5.9), 2.8 (1.5, 5.4), 3.6 (1.9, 6.8), and 3.4 (1.8, 6.5), respectively. Clinical prediction score ranged from 0 to 4.5 with its discrimination concordance (C) statistic of 0.74 (95% CI 0.66, 0.81). Risk stratification classified patients into very low, low, moderate, and high risk groups for SSI when none, one, two, and more than two risk factors were presented with positive likelihood ratio of 1.00, 1.45, 3.32, and 9.28, respectively. A bootstrap demonstrated well calibration and thus good internal validation.

**Conclusions:**

Diabetes, incisional length, fecal contamination, and operative time could be used to predict SSI with acceptable discrimination. This clinical risk prediction should be useful in prediction of SSI. However, external validation should be performed.

**Trial registration:**

ClinicalTrials.gov (ID NCT01659983), registered August 8, 2012

**Electronic supplementary material:**

The online version of this article (10.1186/s13017-018-0186-1) contains supplementary material, which is available to authorized users.

## Background

Superficial surgical site infection (SSI) is common after appendectomy especially in complicated appendicitis (i.e., gangrenous and ruptured) with incidence of 9 to 53% [1, 2]. It increases pain, length of stay, and costs [3]. Risk factors associated with SSI are classified into patient-related, operative-related, and microbe-related factors. It can also be considered according to time of operation as preoperative, intraoperative, and postoperative [4–6].

Accurate prediction of SSI is helpful for management, set up surveillance protocol, and risk stratification for further clinical trial [7]. Risk prediction of SSI after appendectomy had been developed by the National Healthcare Safety Network [8]. The results demonstrated that emergency procedure, gender, hospital bed size > 500, and contaminated wound classification were significant with concordance statistic of 0.70. However, it is difficult to apply into clinical practice due to lack of risk stratification. Other prediction scores of SSI in other contaminated abdominal surgery were available with acceptable discriminative performances [4, 9], but they included heterogeneous groups of patients (i.e., pancreatic, hepatobiliary, and colorectal surgery), which could not be applied to complicated appendicitis [4, 10, 11]. Therefore, this study was conducted to identify risk factors and created clinical prediction score with risk stratification for SSI in complicated appendicitis.

## Methods

### Study design

This study was a part of a randomized controlled trial (RCT) comparing SSI between delayed and primary wound closure in complicated appendicitis [12]. Patients were recruited from two university hospitals (i.e., Ramathibodi and Thammasat University Hospitals) and four regional hospitals (i.e., Lampang Hospital from the North, Chonburi hospital from the Eastern, Surin form the North-eastern, and Pathumthani hospital from the Central). Preoperative inclusion criteria were adult patients, aged ≥ 18 years, who had appendectomy via right lower quadrant abdominal incision. The intraoperative eligible criteria were complicated appendicitis (i.e., gangrenous and rupture). Definition of gangrenous appendicitis was inflamed appendix with necrotic wall (dark, grayish color); ruptured appendicitis was defined as presence of a hole in the appendix, intraoperative rupture, or presence of frank pus. Exclusion criteria were obesity (body mass index (BMI) ≥ 40 kg/m^2^), autoimmune disease, end-stage renal/liver disease, or human immunodeficiency virus. Preoperative and postoperative intravenous antibiotics covering gram-negative and facultative/anaerobic bacilli had been regularly prescribed in all patients until their body temperature (BT) < 37.8 °C for 24 to 48 h, then switched to oral antibiotics for 7 to 10 days as specified in the RCT protocol [12]. The study was reported according to the Transparent Reporting of a Multivariable Prediction Model for Individual Prognosis or Diagnosis (TRIPOD) statement [7]. The original study was approved by the Ethics committee of Ramathibodi Hospital and the collaborating hospitals, in addition to Chonburi hospital for the current study.

### Outcome and predictors

SSI was defined following the Center of Disease Control (CDC) criteria as infection within 30 days that involved skin and subcutaneous tissue, with any of the following: purulent drainage, positive culture of organism isolated from fluid or tissue, or any of the following symptoms/signs: pain or tenderness, localized swelling, redness/heat, or the wound was opened by physician without a positive culture [13]. SSI was assessed before discharging home, at 1-week and 1-month follow-up.

Predictors included in the analysis were patient-related and operative-related factors. Patient-related factors were age, gender, BMI, active smoking, American Society of Anesthesiologists’ (ASA) classification, diabetes, hypertension, duration of symptom before admission, presence of fever at admission (BT ≥ 37.8 °C), white blood cell count, and anemia (hemoglobin ≤ 10 g/dL). Operative-related factors included wound length, subcutaneous fat thickness, type of appendicitis (gangrenous or ruptured), wound contamination with exudative fluid, frank pus, fecal contamination (presence of fecolith or wound contamination with fecal material), presence of phlegmon, operative time, and use of closed suction drain.

### Sample size

As for a rule of thumb of simulation study, at least 10–20 of interested events (i.e., SSI) were required per one predictor in the final model to prevent model optimism [14]. Our data contained 48 SSIs [12]; thus, the final model should include not more than five predictors.

### Statistical analysis

Baseline characteristics of the patients were described. Predictors were compared between SSI and non-SSI using chi-square (or exact test) and Student’s *t* test (or Mann-Whitney *U* test) for categorical and continuous data, respectively. Variables with *p* value less than 0.10 would be included in multivariable analysis. For adjusting purpose, type of wound closure (i.e., delayed primary or primary closure) was included in the multivariable analysis. Forward stepwise logistic regression was applied to identify predictors that significantly associated with SSI (*p* value < 0.05) and thus should be kept in a final parsimonious model.

The coefficients of significant predictors in the parsimonious model were then used to generate risk scores. Then, area under the curve (or concordance (C) statistics) along with its 95% confidence interval (CI) was estimated to quantify discrimination performance. Calibration of the model was assessed using Hosmer-Lemeshow goodness of fit test. Sensitivity, specificity, and positive likelihood ratio (LR) were also estimated. Cutoff point for risk stratification was calibrated according to the LR performance. Posttest probability was finally estimated according to the stratified risk groups.

An internal validation of the risk prediction score was assessed using a bootstrap with 1000 replications [15]. For calibration, the Somer’D coefficient which is an estimate of the correlation between the observed and predicted values of SSI was estimated for all bootstrap data (called *D*_boot_) and derived (original) data (called *D*_org_). A bias was then estimated by subtracting the *D*_org_ with the mean *D*_boot_, lower value reflected less bias and thus better calibration. In addition, the original C statistic was compared to an average C statistic from the bootstraps for discrimination performance. All analysis was performed using STATA version 15.0.

## Results

A total of 607 patients were enrolled from November 2012 to February 2016. Mean age was 45 (standard deviation (SD) 18) years, and 53% of patients were male. Four hundred and nine (76%) had ruptured whereas 148 (24%) had gangrenous appendicitis. A total of 52 patients had SSIs with incidence of 8.7% (95% CI 6.4, 11.0). One patient developed organ space SSI with auto-drainage through incision and was successfully treated non-operatively. Some variables were missing leaving 543 cases with 48 SSIs for the main analysis.

### Model development and validation

Univariable analysis was performed to assess association between each risk factor and SSI, see Table 1. For ease of application, continuous variables were categorized into two groups based on 75 percentiles except operative time that was categorized according to National Nosocomial Infections Surveillance (NNIS) [16]. The cut points were 25.7 kg/m^2^, 48 h, 7 cm, 4.5 cm, and 75 min for BMI, duration symptoms, incisional length, subcutaneous fat thickness, and operative time, respectively. Among 19 predictors, eight had *p* <  0.10 including BMI, diabetes, duration of symptoms, appendicitis classification, incisional length, subcutaneous fat thickness, fecal contamination, and length of operative time. Stepwise logistic regression identified four predictors in the parsimonious model, i.e., presence of diabetes, incisional length, fecal contamination, and operative time > 75 min, see Table 2. The risk prediction equation was written as

**Table 1:** Risk factors associated with superficial surgical site infection in complicated appendicitis

Risk factors	No SSI	SSI	*P* value
Patient-related			
Gender, number (%)			
Male	291 (90.9)	29 (9.1)	0.773
Female	255 (91.7)	23 (8.3)	
Age, year, mean (SD)	45.5 (0.78)	44.4 (2.31)	0.674
BMI, kg/m^2^, mean (SD)	23.3 (0.19)	24.5 (0.48)	0.023
Smoking, number (%)			
Yes	85 (90.4)	9 (9.6)	0.693
No	460 (91.2)	43 (8.6)	
ASA classification, number (%)			
Class I	319 (92.7)	25 (7.3)	0.290
Class II	151 (88.8)	19 (11.2)	
Class III	65 (90.1)	7 (9.7)	
Class IV	5 (83.3)	1 (16.7)	
Diabetes, number (%)			
Yes	40 (78.4)	11 (21.6)	0.001
No	502 (92.5)	41 (7.5)	
Hypertension, number (%)			
Yes	104 (91.2)	10 (8.8)	0.989
No	439 (91.3)	42 (8.7)	
Duration of symptoms, hours, median (IQR)	24 (14, 48)	24 (24, 48)	0.055
Presence of fever (≥ 37.8 °C), number (%)			
Yes	284 (92.2)	24 (7.8)	0.389
No	258 (90.2)	28 (9.8)	
White blood cell count, cell/mm^3^, mean (SD)	15,563 (4998)	16,658 (4551)	0.130
Hct < 30%, number (%)			
Yes	25 (92.6)	2 (7.4%)	0.821
No	517 (91.3)	49 (8.7%)	
Operative-related			
Appendicitis severity classification, number (%)			
Gangrene	141 (97.2)	4 (2.8)	0.004
Ruptured	405 (89.4)	48 (10.6)	
	No SSI	SSI	*P* value
Wound			
Incisional length, cm, mean (SD)	5.6 (2.24)	6.9 (2.62)	< 0.001
Subcutaneous fat thickness, cm, mean (SD)	3.0 (2.00)	3.7 (2.20)	0.033
Visible wound contamination, number (%)			
Exudative fluid			
Yes	147 (88.6)	19 (11.6)	0.139
No	399 (92.4)	33 (7.6)	
Purulent fluid			
Yes	202 (90.2)	22 (9.8)	0.450
No	344 (92.0)	30 (8.0)	
Fecal contamination, number (%)			
Yes	143 (85.1)	25 (14.9)	0.001
No	378 (93.8)	25 (6.2)	
Operative time (minutes), number (%)			
≤ 75	129 (83.8)	25 (16.2)	< 0.001
> 75	412 (93.9)	27 (6.2)	
Used of closed suction drain, number (%)			
Yes	109 (93.2)	8 (6.8)	0.413
No	433 (90.8)	44 (9.2)	

*ASA* American Society of Anesthesiologists, *BMI* body mass index, *SD* standard deviation, *SSI* superficial surgical site infection

**Table 2:** Risk factors of superficial surgical site infection: a multiple logistic regression

Variables	Coefficient	SE	*P* value	OR (95% CI)	Score
Diabetes					
Yes	0.96	0.42	0.021	2.6 (1.2, 5.9)	1
No					0
Operative time					
≤ 75 min	1.22	0.33	< 0.001	3.4 (1.8, 6.5)	1.2
> 75 min					0
Fecal contamination					
Yes	1.27	0.33	< 0.001	3.6 (1.9, 6.8)	1.3
No					0
Incisional length					
≤ 7 cm	1.03	0.33	0.002	2.8 (1.5, 5.4)	1
> 7 cm					0
Total					0–4.5

*CI* confidence interval, *SE* standard error, *OR* odds ratio

$$ \ln \left[\frac{P}{1-P}\right]=-3.70+0.96\ast (diabetes)+1.22\ast \left( operative\ time\right)+1.27\ast \left( fecal\ contamination\right)+1.03\ast \left( incisional\ length\right) $$

**Fig. 1:**
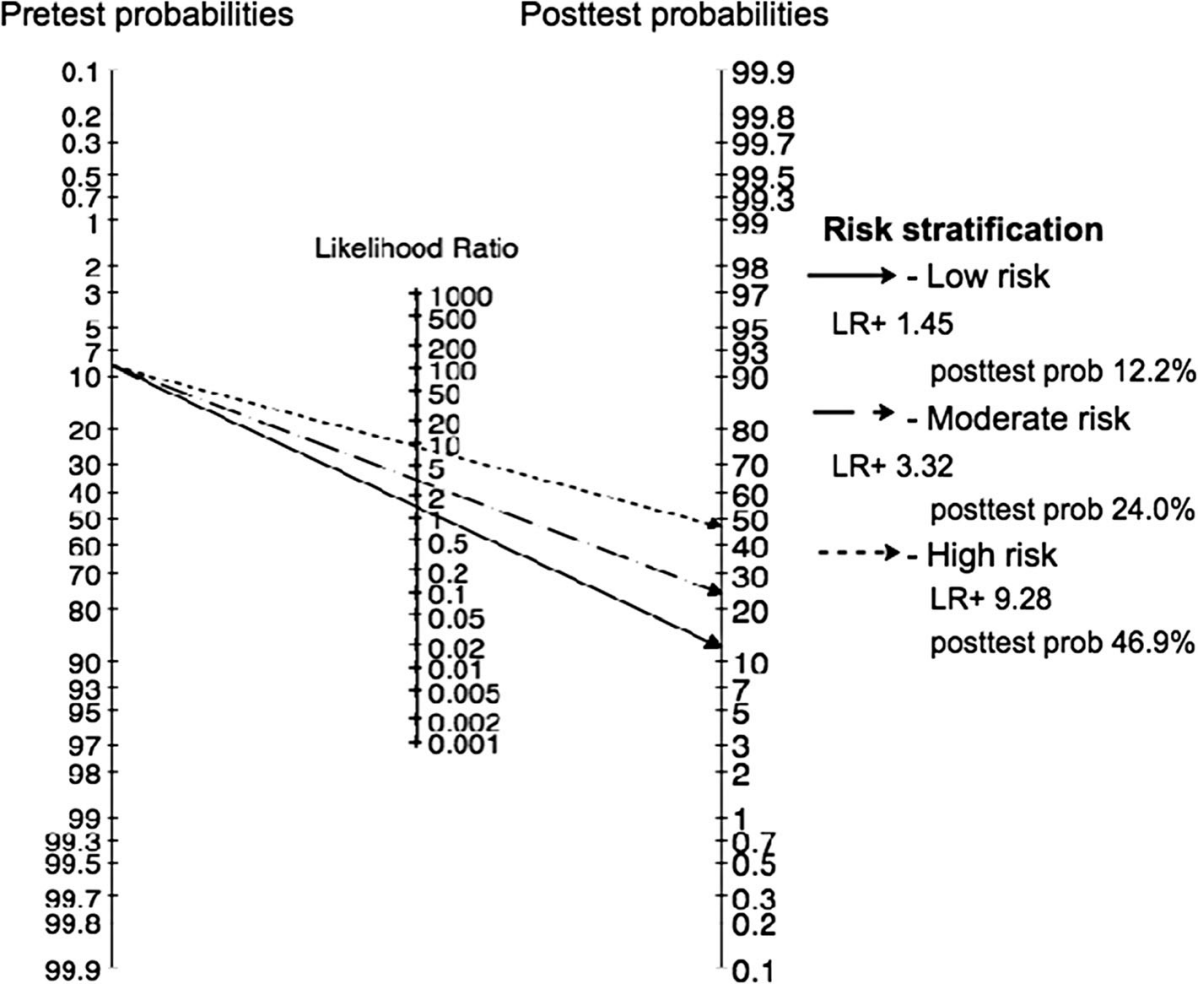
Fagan’s plot for risk prediction score for superficial surgical site infection in complicated appendicitis

The C statistics of this model was 0.74 (95% CI 0.66, 0.81) indicating acceptable discrimination of SSI from non-SSI. Hosmer-Lemeshow goodness of fit test was applied and demonstrated good calibration of the model (chi-square = 4.42, *p* value = 0.491), see Additional File 1: Table S1. Coefficients of significant predictors were used to calculate prediction score. Diabetes and incision length were weighted 1; operative time and fecal contamination were weighted 1.2 and 1.3, respectively. Adding them up resulted in risk score ranged from 0 to 4.5, see Table 2. Cutoff points were identified based on positive LR of each distinct score from ROC curve analysis. The score was classified as 0, 1–1.3, 2–2.5, and > 2.5 for very low, low, moderate, and high risk groups, respectively. Sensitivity, specificity, positive LR, and posttest probabilities were demonstrated in Table 3. Fagan’s nomogram was constructed, and posttest probabilities were calculated based on incidence of SSI in this study which was 8.7% (95% CI 6.4, 11.0), see Fig. 1 [12]. Patients had probabilities of SSI of 12.2 (95% CI 9.0, 15.5), 24.0 (95% CI 18.5, 29.5), and 46.9 (95% CI 38.8, 53.9) for low, moderate, and high risk groups, respectively.

**Table 3:** Risk stratification of prediction values of scoring system

Risk classification	Scores	Outcome		Sensitivity (%)	Specificity (%)	Correctly classify (%)	LR+^a^	Posttest probability (95% CI)^b^
		SSI	No SSI					
Low	1–1.3	21	411	85.4	41.2	8.84	1.453	12.2 (9.0, 15.5)
Moderate	2–2.5	18	74	56.3	83.0	80.7	3.315	24.0 (18.5, 29.5)
High	> 2.5	9	10	18.8	98.0	91.0	9.281	46.9 (38.8, 53.9)

Coefficient of constant term in the final model (− 3.70) was omitted for easier understanding and interpretation

^a^Compared to patients with very low risk (score = 0)

^b^Based on pretest probability, incidence of SSI, of 8.7%

*CI* confidence interval, *LR* likelihood ratio, *SSI* superficial surgical site infection

A bootstrap with 1000 replications was performed to assess internal validation, which yielded estimated *D*_org_ and *D*_boot_ coefficients of 0.476 and 0.500 (95% CI 0.496, 0.506) for the derivative and bootstrap models, respectively. The average bias was only − 0.025 (95% CI − 0.030, − 0.019), suggesting good calibration. The bootstrap C statistics was 0.750 (95% CI 0.748, 0.753), with a bias of − 0.012 (95% CI − 0.015, − 0.010).

## Discussion

Our study demonstrated that presence of diabetes, incisional length > 7 cm, fecal contamination, and operative time > 75 min were significantly associated with superficial SSI after open appendectomy via right lower quadrant abdominal incision in complicated appendicitis. A risk prediction score was then constructed with good performances, i.e., well calibrated and good discrimination with C statistic of 0.74 (95% CI 0.66, 0.81). The score was classified into four groups: very low, low, moderate, and high risk with positive LR of 1.453, 3.315, and 9.281 for the latter three groups, respectively.

All predictors included in the model were consistently demonstrated to be associated with SSI in other studies (i.e., diabetes [17, 18], incisional length [19], fecal contamination [18, 19], and operative time [9, 20]). Both preoperative and intraoperative factors play roles in SSI. Higher BMI [21, 22] and subcutaneous fat thickness [23] had been shown to be associated with SSI in other studies but they were not and thus excluded from the model during a selection process in this study.

About 11.8% of total patients whose data were missing were dropped out during development of risk prediction model. Characteristics of patients between complete and incomplete data were explored, distributions of BMI, diabetes, hypertension, and operative time were quite different, but only BMI was significant, see Additional file 2: Table S2. Multiple imputation by chain equations with 10 imputations was attempted to fill in missing data. The final risk prediction model was constructed, and their coefficients were not much different comparing with using only complete data, see Additional file 3: Table S3. Bias from using only complete data should be less likely.

Laparoscopic appendectomy has been associated with less incidence of SSI than open surgery and has replaced open appendectomy in some countries [24]. However, it required much experienced with high cost. Therefore, it is still less performed in developing country than developed country, and open appendectomy is still the standard of care [25].

### Clinical application

Applying our model is simplified by counting number of four risk factors (i.e., diabetes, operative time > 75 min, fecal contamination, and incision length > 7 cm). According to the risk stratification scores, patient with 0, 1, 2, and > 2 risk factors will be respectively classified into very low, low, moderate, and high risk groups with positive LR for low, moderate, and high scores of 1.4, 3.3, and 9.2, in which positive LR of 5 to 10 could moderately shift pre- to posttest probability as for EBM working group recommendation [26]. To calculate posttest probabilities of SSI, Fagan’s nomogram could be used or to estimate posttest probability of SSI occurrence according to risk prediction score [27]. For instance, if patient has history of diabetes, operative time > 75 mins, and fecal contamination, she/he will be classified as high risk group (three factors); posttest probability of SSI for this patient is about 46%. If patient does not have history of diabetes, but has fecal contamination and incision length > 7 cm, this patient will be classified as moderate risk and thus her/his posttest probability is about 20–30%.

The results from previous randomized controlled trial [12] demonstrated that delayed primary wound closure had no benefit over primary wound closure in normal risk patients and primary wound closure should be done with risk of SSI of 8.7% (95% CI 6.4, 11.2). We further suggested closing the wound primarily in the low risk group with risk of SSI of 12.3 (95% CI 9.0, 15.5). However, in moderate and high risk groups, primarily closing the wound should be done with caution. Other wound interventions such as wound edge protectors [28], subcutaneous wound drainage [29], or daily wound probing [30] should be considered to apply.

Early detection and treatment of SSI is also important to reduce impact of SSI to a patient, which includes pain, isolation, insecurity, and costs [31]. Knowing risk of SSI can help physician educate, inform, and arrange appropriate infection surveillance protocol. Patients should be advised about symptoms and signs of SSI before discharging home. Patient information sheets or self-assessment questionnaires may be useful [32].

The model was derived with standard methodological analysis including 4 markedly significant variables. Risk stratification was also easy to apply by just counting occurring variables at the end of operation. The internal validation also demonstrated good discrimination and calibration. The final prediction model contained 4 variables that should not result in model optimism with 48 events of SSI as for recommendation by TRIPOD [7]. An internal validation was performed using a bootstrapping technique, which was more appropriated than splitting data when a number of event of interest was small [7]. However, external validation and impact of applying the model to outcomes should be done in the future [33].

## Conclusions

The risk prediction score of SSI has been developed containing diabetes, operative time, fecal contamination, and incisional length. The score internally performed well with good calibration and discrimination. This can be ease of use in clinical practice. However, the score should also be further externally validated.

## Additional files

Additional file 1: **Table S1.** Hosmer-Lemeshow goodness of fit. Table describing details of Hosmer-Lemeshow goodness of fit. (DOCX 17 kb)

Additional file 2: **Table S2.** Describe distribution of variables between complete and incomplete data. Baseline characteristics comparison between cases with complete and incomplete data. (DOCX 21 kb)

Additional file 3: **Table S3.** Risk factors of superficial surgical site infection based on imputed data. Significant variables with their estimated coefficiencies in the final parsimonious model estimated from imputed data. (DOCX 16 kb)

## Abbreviations

ASA: American Society of Anesthesiologists
BMI: Body mass index
BT: Body temperature
C: Concordance
CDC: Center of Disease Control
CI: Confidence interval
LR: Likelihood ratio
NNIS: National Nosocomial Infections Surveillance
ROC: Receiver operating characteristic
SD: Standard deviation
SSI: Superficial surgical site infection
TRIPOD: Transparent Reporting of a Multivariable Prediction Model for Individual Prognosis or Diagnosis
