# Supplementary material for: Clinical prediction score for superficial surgical site infection after appendectomy in adults with complicated appendicitis
Source: World J Emerg Surg. 2018 Jun 18;13:23. doi: 10.1186/s13017-018-0186-1 (PMC6006790; doi:10.1186/s13017-018-0186-1)
Supplement: Supplementary file 1 — Table S1. Hosmer-Lemeshow goodness of fit. Table describing details of Hosmer-Lemeshow goodness of fit. (DOCX 17 kb) [file 13017_2018_186_MOESM1_ESM.docx]

Table S1. Hosmer-Lemeshow goodness of fit

| **Group** | **Probabilities** | **Obs_1** | **Exp_1** | **Obs_0** | **Exp_0** | **Total** |
| --- | --- | --- | --- | --- | --- | --- |
| 3 | 0.0241 | 7 | 5.1 | 204 | 205.9 | 211 |
| 4 | 0.0606 | 1 | 0.8 | 12 | 12.2 | 13 |
| 6 | 0.0771 | 9 | 8.9 | 112 | 112.1 | 121 |
| 7 | 0.0811 | 4 | 7.1 | 83 | 79.9 | 87 |
| 8 | 0.1534 | 0 | 0.8 | 5 | 4.2 | 5 |
| 9 | 0.1986 | 11 | 12.5 | 54 | 52.5 | 65 |
| 10 | 0.4559 | 16 | 12.9 | 25 | 28.1 | 41 |

number of observations = 543

number of groups = 7

Hosmer-Lemeshow chi2(5) = 4.42

Prob > chi2 = 0.4909
