# Supplementary material for: Clinical prediction score for superficial surgical site infection after appendectomy in adults with complicated appendicitis
Source: World J Emerg Surg. 2018 Jun 18;13:23. doi: 10.1186/s13017-018-0186-1 (PMC6006790; doi:10.1186/s13017-018-0186-1)
Supplement: Supplementary file 2 — Table S2. Describe distribution of variables between complete and incomplete data. Baseline characteristics comparison between cases with complete and incomplete data. (DOCX 21 kb) [file 13017_2018_186_MOESM2_ESM.docx]

Table S2. Describe distribution of variables between complete and incomplete data

| Risk factors | Complete data  (n = 543) | Missing data  (n = 64) | *P* value |
| --- | --- | --- | --- |
| Patient-related |  |  |  |
| Gender, number (%)  Male  Female, number | 291 (54)  252 (46) | 33 (52)  31 (48) | 0.758 |
| Age, year, mean (SD) | 46 (18) | 42 (17) | 0.149 |
| BMI, kg/m^2^, mean (SD) | 23.5 (4.4) | 22.2 (3.7) | 0.027 |
| Smoking, number (%)  Yes  No | 84 (15)  459 (85) | 12 (19)  51 (81) | 0.462 |
| ASA classification, number (%)  Class I  Class II  Class III  Class IV | 310 (58)  155 (29)  69 (13)  5 (1) | 41 (66)  17 (27)  3 (5)  1 (2) | 0.267 |
| Diabetes, number (%)  Yes  No | 49 (9)  494 (91) | 2 (3)  58 (97) | 0.133 |
| Hypertension, number (%)  Yes  No | 108 (20)  435 (80) | 6 (10)  54 (90) | 0.063 |
| Duration of symptoms, hours, median (IQR) | 24 (15, 48) | 24 (12, 48) | 1.000 |
| Presence of fever (≥ 37.8 °C), number (%)  Yes  No | 302 (56)  237 (44) | 34 (56)  27 (44) | 0.965 |
| White blood cell count, cell/mm^3^, mean (SD) | 15741 (5016) | 14983 (4482) | 0.253 |
| Hct < 30%, number (%)  Yes  No | 34 (6)  508 (94) | 6 (10)  56 (90) | 0.307 |
| Operative-related |  |  |  |
| Appendicitis severity classification, number (%)  Gangrene  Ruptured | 128 (24)  415 (76) | 20 (31)  44 (69) | 0.176 |

Supplement Table 2. Describe distribution of variables between complete and incomplete data (continued)

| Risk factors | Complete data  (n = 543) | Missing data  (n = 64) | *P* value |
| --- | --- | --- | --- |
| Wound  Incisional length, cm, mean (SD)  Subcutaneous fat thickness, cm, mean (SD)  Visible wound contamination, number (%)  Exudative fluid  Yes  No  Purulent fluid  Yes  No | 5.8 (2.3)  3.1 (2.1)  152 (28)  391 (72)  208 (38)  335 (62) | 5.1 (1.5)  2.4 (1.4)  16 (25)  48 (75)  18 (28)  46 (72) | 0.074  0.067  0.613  0.111 |
| Fecal contamination, number (%)  Yes  No | 160 (29)  383 (71) | 9 (26)  26 (74) | 0.636 |
| Operative time (minutes), number (%)  ≤ 75  > 75 | 147 (27)  396 (73) | 10 (17)  49 (83) | 0.093 |
| Used of closed suction drain, number (%)  Yes  No | 109 (20)  433 (80) | 11 (18)  50 (82) | 0.700 |

ASA, American Society of Anesthesiologists’; BMI, body mass index; cm, centimeter; kg, kilogram; SD, standard deviation; SSI, superficial surgical site infection
