# Supplementary material for: Clinical prediction score for superficial surgical site infection after appendectomy in adults with complicated appendicitis
Source: World J Emerg Surg. 2018 Jun 18;13:23. doi: 10.1186/s13017-018-0186-1 (PMC6006790; doi:10.1186/s13017-018-0186-1)
Supplement: Supplementary file 3 — Table S3. Risk factors of superficial surgical site infection based on imputed data. Significant variables with their estimated coefficiencies in the final parsimonious model estimated from imputed data. (DOCX 16 kb) [file 13017_2018_186_MOESM3_ESM.docx]

Table S3. Risk factors of superficial surgical site infection based on imputed data

| Variables | Coefficient | SE | *P* value | OR (95%CI) | Score |
| --- | --- | --- | --- | --- | --- |
| Diabetes  Yes  No | 1.19 | 0.41 | 0.003 | 3.29 (1.5, 7.2) | 1.1  0 |
| Operative time  ≤ 75 minutes  > 75 minutes | 1.11 | 0.31 | < 0.001 | 3.04 (1.6, 5.6) | 1.1  0 |
| Fecal contamination  Yes  No | 1.27 | 0.32 | < 0.001 | 3.6 (1.9, 6.6) | 1.3  0 |
| Incisional length  ≤ 7 cm  > 7 cm | 0.92 | 0.32 | 0.005 | 2.5 (1.3, 4.7) | 0.9  0 |
| Total |  |  |  |  | 0- 4.4 |

CI, confidence interval; OR, odd ratios; SE, standard error
